# Private Equity in Orthopaedics: An Analysis of Practice Acquisitions, 2016 to 2023

**DOI:** 10.2106/JBJS.OA.26.00128

**Published:** 2026-10-15

**Authors:** Kathryn R. Segal, Stephen Wendolowski, Megha Reddy, Phillip Schmitt, Raymond Y. Hsu, Yashaswini Singh

**Affiliations:** 1 Department of Orthopaedic Surgery, Warren Alpert Medical School, Brown University, Providence, Rhode Island; 2 Department of Health Services, Policy & Practice, School of Public Health, Brown University, Providence, Rhode Island

## Abstract

**Introduction:**

Private equity (PE) has had an increasing influence in health care, including procedural subspecialties such as orthopaedic surgery. No studies yet have characterized the orthopaedic practices that are acquired by PE firms.

**Methods:**

This cross-sectional study analyzed PE acquisitions of orthopaedic practices in the United States between January 1, 2016, and December 31, 2023. A combination of PitchBook and manual web searches was used to identify PE acquisitions. The Medicare Data on Provider Practice and Specialty were used to identify Tax Identification Numbers of practices and the unique providers of each practice at time of affiliation.

**Results:**

Eighty-nine orthopaedic practices were identified from 2016 to 2023, accounting for 4.8% of orthopaedists by 2023. More than three-quarters of acquisitions took place from 2021 to 2023 (77.5%). Nearly half of acquisitions were in the South (47.2%). At time of acquisition, 36.6% of providers in the practices were surgeons, 10.0% were nonsurgical physicians, and 53.4% were ancillary providers, such as advanced practice providers and therapists. The most represented subspecialties included sports (24.8%) and arthroplasty (18.2%), and the least represented were pediatrics (0.7%) and tumor (0.3%).

**Conclusion:**

PE involvement in orthopaedics skyrocketed in 2021. Practices targeted by PE firms typically included a multidisciplinary mix of providers, more than half of whom were nonphysician adjuncts, with a geographic predilection for the South. Orthopaedic subspecialties with historically higher reimbursements and outpatient volumes were overrepresented in PE-acquired practices. Future work should examine the resulting influence of PE in orthopaedics, including provider turnover, practice patterns, and clinical outcomes.

**Level of Evidence:**

Level III. See Instructions for Authors for a complete description of levels of evidence.

## Introduction

Private equity (PE) continues to expand its interest in health care with a growing investment in physician practices^1,2^. Health care is appealing to investors given its stable demand outside of economic factors. PE firms leverage private capital combined with debt to purchase target companies, and through active management seek to improve profitability. For physician practices, PE employs a “platform and add-on” strategy by purchasing a large established practice (platform) and acquiring smaller practices (add-on) to increase market share and develop economies of scale. The PE firm may operate through a management services organization (MSO) that provides administrative support to physician practices, serving as an intermediary between the firm and physicians^3,4^. Eventually, through active management and further practice acquisitions, the PE firm can “exit” investments by reselling the acquired practice for a higher valuation. The goal is to sell the company at a higher valuation in short investment horizons, generally after 3 to 7 years^5^.

PE initially entered the U.S. healthcare market with purchases of nursing homes and hospitals in the early 2000s. Research has shown that PE investments in nursing homes and hospitals are associated with reductions in staffing with potential for patient harm^6,7^. However, since 2015 there has been a dramatic shift to acquiring specialized physician practices^1^. Cardiology, dermatology, gastroenterology, and ophthalmology have all been strongly influenced by PE^1,2,8-13^. Research shows that PE investments in these fields have been associated with higher spending and utilization, and changes to workforce composition^8,14,15^. While PE can also bring operational efficiencies and capital infusions that can facilitate technological investments to improve patient care, these theoretical benefits have not been examined in research to date.

Recently, PE has increased its presence in orthopaedics through acquisition of private practices. Prior studies have demonstrated increases in PE involvement at the end of the 2010s^16-20^. From an investor perspective, orthopaedics is an attractive investment given the increased utilization of ambulatory surgical centers (ASCs), where practices earn the additional facility fee; the ancillary revenue streams, such as physical and occupational therapies and musculoskeletal imaging; and an aging population driving growth. There is also significant opportunity for consolidation of the orthopaedic market. In 2018, an American Academy of Orthopaedic Surgeons (AAOS) survey demonstrated 51% of orthopaedists belonged to private or multispecialty practices with less than 10 physicians^21^. As of 2024, 54% of orthopaedists were in private practice, making it attractive to financial investors and other corporate entities seeking returns through consolidation^22^.

Despite growing attention to the role of PE in orthopaedics, there remains a paucity in the literature characterizing the acquisition of orthopaedic practices. The present study aims to characterize PE involvement in orthopaedics and describe the practices and types of providers targeted.

## Methods

### Identifying PE Acquisitions

This cross-sectional study examined PE acquisitions of orthopaedic practices between January 1, 2016, and December 31, 2023. Acquisition data were obtained from PitchBook, a financial database, and were manually verified and expanded using publicly available information, including press releases and clinic websites. PE acquisitions are defined as leveraged buyouts of patient-facing clinics financially backed by a PE sponsor. This approach follows that of prior studies examining PE in health care.^9,11-15,23-25^

## Identifying Providers and Specialties

Following previous research, we used the Medicare Data on Provider Practice and Specialty (MD-PPAS), Tax Identification Numbers (TINs), and National Provider Identifiers (NPIs) to define practices and affiliated providers^13,15,25^. All unique NPIs that billed under a specific TIN during the year of acquisition were included. Provider specialty was determined by specialty listed in MD-PPAS (from the Medicare Provider Enrollment, Chain, and Ownership System [PECOS] database). The respective PECOS code associated with each specialty is listed in Supplemental Table 1. Surgeons were defined as all providers listed as orthopaedic surgery, as well as hand surgery, neurosurgery, plastic and reconstructive surgery (PRS), and podiatry. Nonsurgeon physicians included physical medicine and rehabilitation (PM & R), primary care sports medicine, pain medicine, neurology, anesthesia, diagnostic and interventional radiology, psychiatry and psychology, internal medicine (IM) and IM-subspecialties, family medicine, and emergency medicine. Nonphysician providers included advanced practice providers (APPs, physician assistants, and nurse practitioners), physical therapists, occupational therapists, and speech-language pathologists.

Surgical subspecialty was determined based on the listed subspecialty on practice websites and/or fellowship training. If an orthopaedist did not complete a fellowship and no clear subspecialty was identified, they were classified as general orthopaedics. For comparisons of subspecialties among practices, we combined 3 subspecialties of orthopaedists with other surgical providers. “Hand surgeons” included (1) orthopaedists by MD-PPAS who practiced hand based on subspecialty review, (2) hand surgeons by MD-PPAS, and (3) PRS surgeons by MD-PPAS who practice hand for orthopaedic practices. “Spine surgeons” included (1) orthopaedists by MD-PPAS who practiced spine based on subspecialty review and (2) neurosurgeons by MD-PPAS who practiced spine for orthopaedic practices. “Foot and ankle surgeons” included (1) orthopaedists by MD-PPAS who practiced foot and ankle based on subspecialty review, and (2) podiatrists by MD-PPAS who worked for orthopaedic practices.

MD-PPAS data were available from years 2014 to 2022. For acquisitions that occurred in 2023, provider composition was based upon TIN and NPI data from the 2022 data set. Provider demographics including age, gender, and race were not included in analyses.

### Geographic Penetration

Geographic penetration of PE-affiliated orthopaedic surgeons used the 2022 MD-PPAS data set but similarly included practices that had been acquired through 2023. All providers classified as orthopaedic surgery were included. PE orthopaedists were identified if they billed to a PE-acquired TIN. The state was determined by the individual orthopaedist’s listed state in MD-PPAS, which was not necessarily the state of the practice with which the surgeon was affiliated.

The study was conducted with an Institutional Review Board waiver of informed consent as no individual-level data were used. The study follows the Strengthening the Reporting of Observational Studies in Epidemiology (STROBE)–reporting guidelines. Statistical analyses were performed in Stata (Version17.0, StataCorp). Figures were generated using Microsoft Excel and MapChart.net.

## Results

Eighty-nine practice acquisitions were identified from 2016 to 2023 (Table 1, Fig. 1). Yearly acquisitions ranged from 1 in 2016 to 28 in 2022. There was a notable increase from 5 acquisitions in 2020 to 23 in 2021. 77.5% of acquisitions occurred from 2021 to 2023 (69/89). The median size of an acquired practice was 22 providers: 10 surgeons, 2 nonsurgical physicians, and 10 nonphysician providers. Nearly half of acquired practices were in the South (47.2%, 42/89), while only 12.4% were in the West (11/89).

**Table 1:** Characteristics of Orthopaedic Practices During the Year of PE Affiliation

	No. of Practices*	Practice Size†	No. of Orthopaedic Surgeons	No. of Other Surgeons‡	No. of Nonsurgeon Physicians§	No. of APPs and Therapists
	N (%)	Median (IQR)	Median (IQR)	Median (IQR)	Median (IQR)	Median (IQR)
All	89	22 (12, 46)	8 (4, 14)	1 (0, 3)	2 (0, 5)	10 (4, 23)
Region						
Northeast	17 (19.1)	25 (21, 43)	11 (5, 13)	1 (0, 3)	2 (1, 7)	13 (8, 18)
Midwest	19 (21.4)	16 (10, 57)	6 (4, 15)	1 (1, 4)	1 (0, 5)	8 (4, 26)
South	42 (47.2)	22.5 (11, 45)	8 (5, 13)	1 (0, 2)	1 (0, 4)	10 (4, 23)
West	11 (12.4)	33 (13, 95)	8 (4, 18)	3 (1, 5)	4 (2, 11)	8 (1, 60)
Year of acquisition						
2016	1 (1.1)	31	10	0	8	13
2017	4 (4.5)	50.5 (28.5, 102.5)	13 (6, 28.5)	3.5 (1.5, 9)	8 (6.5, 13.5)	27.5 (14.5, 51.5)
2018	2 (2.3)	23.5 (2, 45)	6 (1, 11)	1 (0, 2)	0.5 (0, 1)	16 (0, 32)
2019	8 (9.0)	51.5 (6, 74)	13.5 (2.5, 18.5)	1.5 (0, 3)	3.5 (0.5, 7.5)	27 (2.5, 51)
2020	5 (5.6)	19 (15, 24)	6 (5, 14)	0 (0, 1)	2 (2, 3)	8 (1, 11)
2021	23 (25.8)	20 (9, 37)	7 (3, 13)	1 (0, 3)	1 (0, 3)	8 (3, 16)
2022	28 (31.5)	23 (18, 41.5)	8 (5, 13)	1 (0, 4)	2.5 (0.5, 5.5)	10 (7.5, 24.5)
2023	18 (20.2)	16.5 (9, 43)	7.5 (4, 13)	1 (0, 2)	1 (0, 4)	8 (3, 19)

APP = advanced practice provider, IQR = interquartile range, MD-PPAS = Medicare Data on Provider Practice and Specialty, NPI = National Provider Identifiers, PE = private equity, and TIN = Tax Identification Number.

Source: Authors’ calculation of data from PitchBook, MD-PPAS, and manual data collection.

* Practices were defined by individual TINs.

† Practice size is based upon the number of unique NPIs billed under a practice’s TIN during the year of acquisition.

‡ The number of surgeons classified as hand surgery, plastics and reconstructive surgery, neurosurgery, and podiatry in the MD-PPAS data set.

§ Includes physicians classified as physical medicine and rehabilitation, primary care sports medicine, pain medicine, neurology, anesthesia, radiology, psychiatry and psychology, internal medicine and specialties, family medicine, and emergency medicine.

**Fig. 1:**
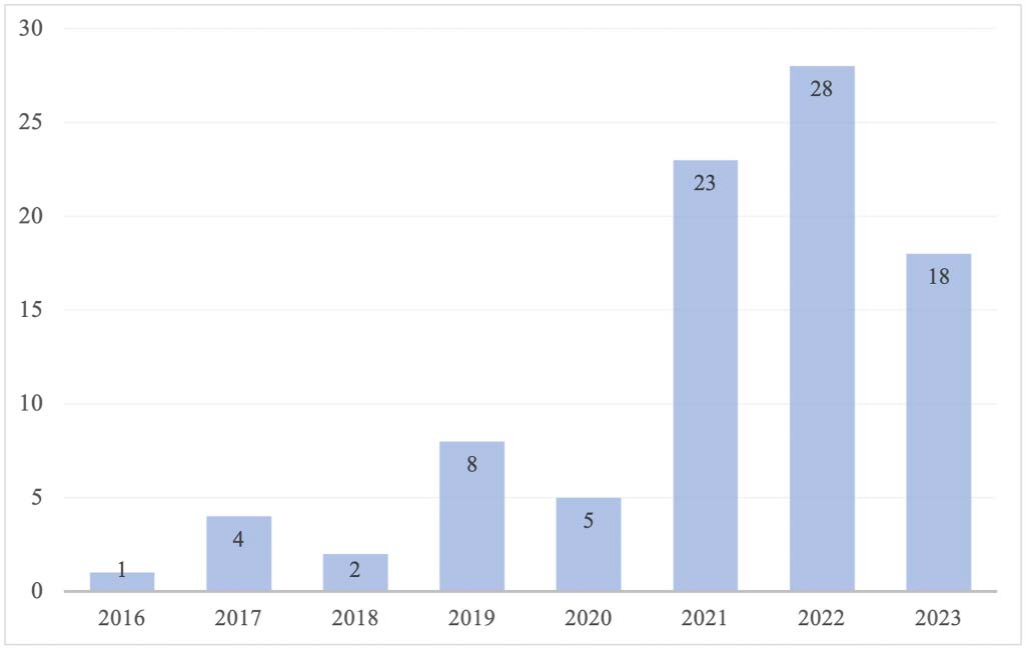
The number of new orthopaedic practices affiliated with PE firms by year.

Only 36.6% of the providers in acquired practices were surgeons, and 10.0% were nonsurgical physicians (Fig. 2, Supplemental Table 2). 53.4% of providers were APPs and therapists. Among 1,209 surgeons, the most represented subspecialties were sports (24.8%), arthroplasty (18.2%), hand (14.6%), and spine and foot and ankle (13.6% each). The least represented specialties were tumor (0.3%), pediatrics (0.7%), and trauma (2.7%). Among 330 nonsurgical physicians, the most represented were PM&R (34.8%), primary care sports medicine (17.3%), and pain management (14.3%). More than half of the nonphysician providers were APPs (51.7%), followed by physical therapists (41.6%) and occupational therapists (6.6%).

**Fig. 2:**
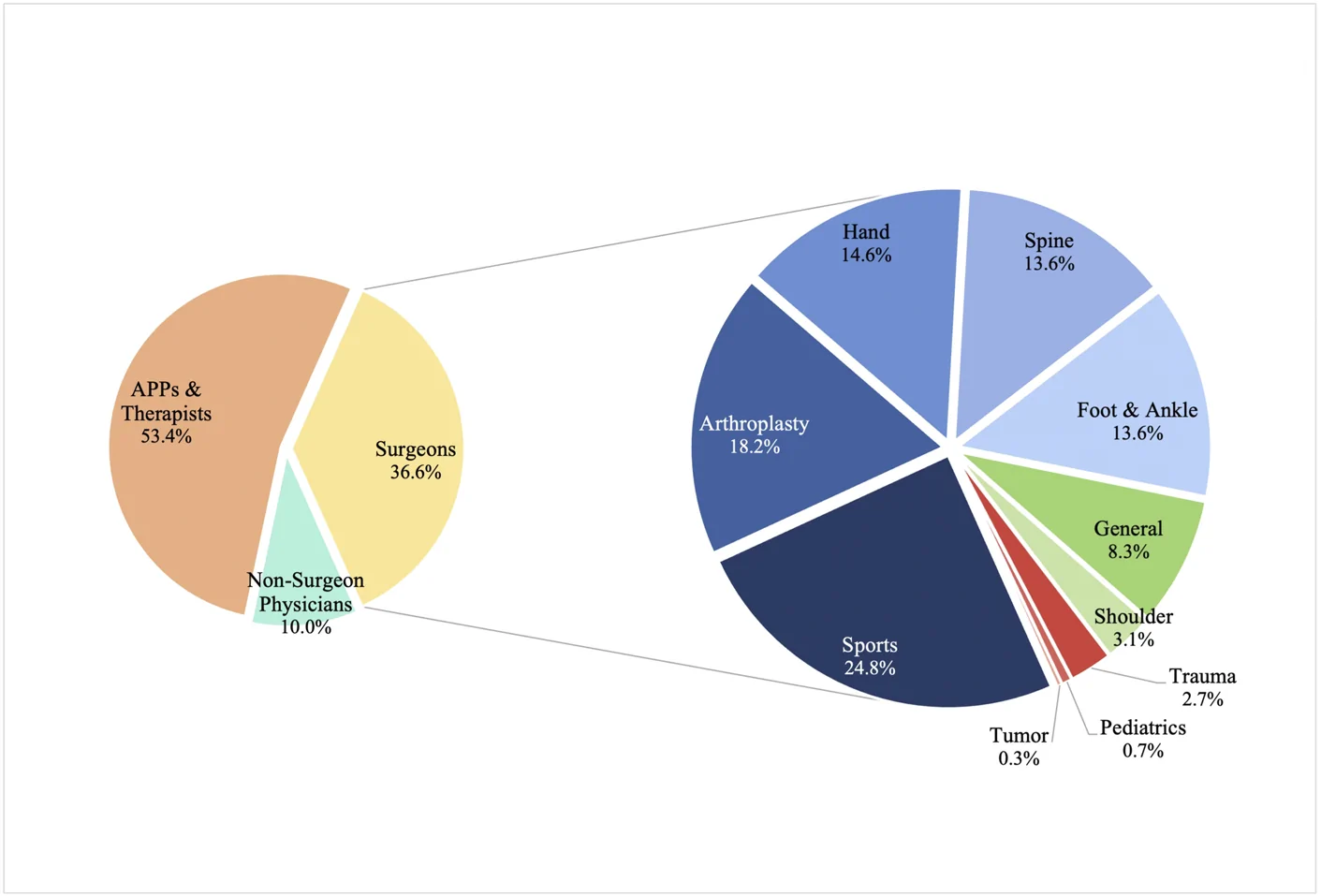
Provider type and surgical subspecialty breakdown of providers at time of PE affiliation.

By 2023, 4.8% of orthopaedists in the United States were practicing in a PE-affiliated group (Fig. 3, Supplemental Table 3). The highest penetrated state was Mississippi (30.1% of orthopaedists), followed by Connecticut (17.5%). Nineteen states, including the District of Columbia, did not have any. Table 2 lists the largest 8 PE firms, by number of practices, with their geographic location and practice compositions.

**Fig. 3:**
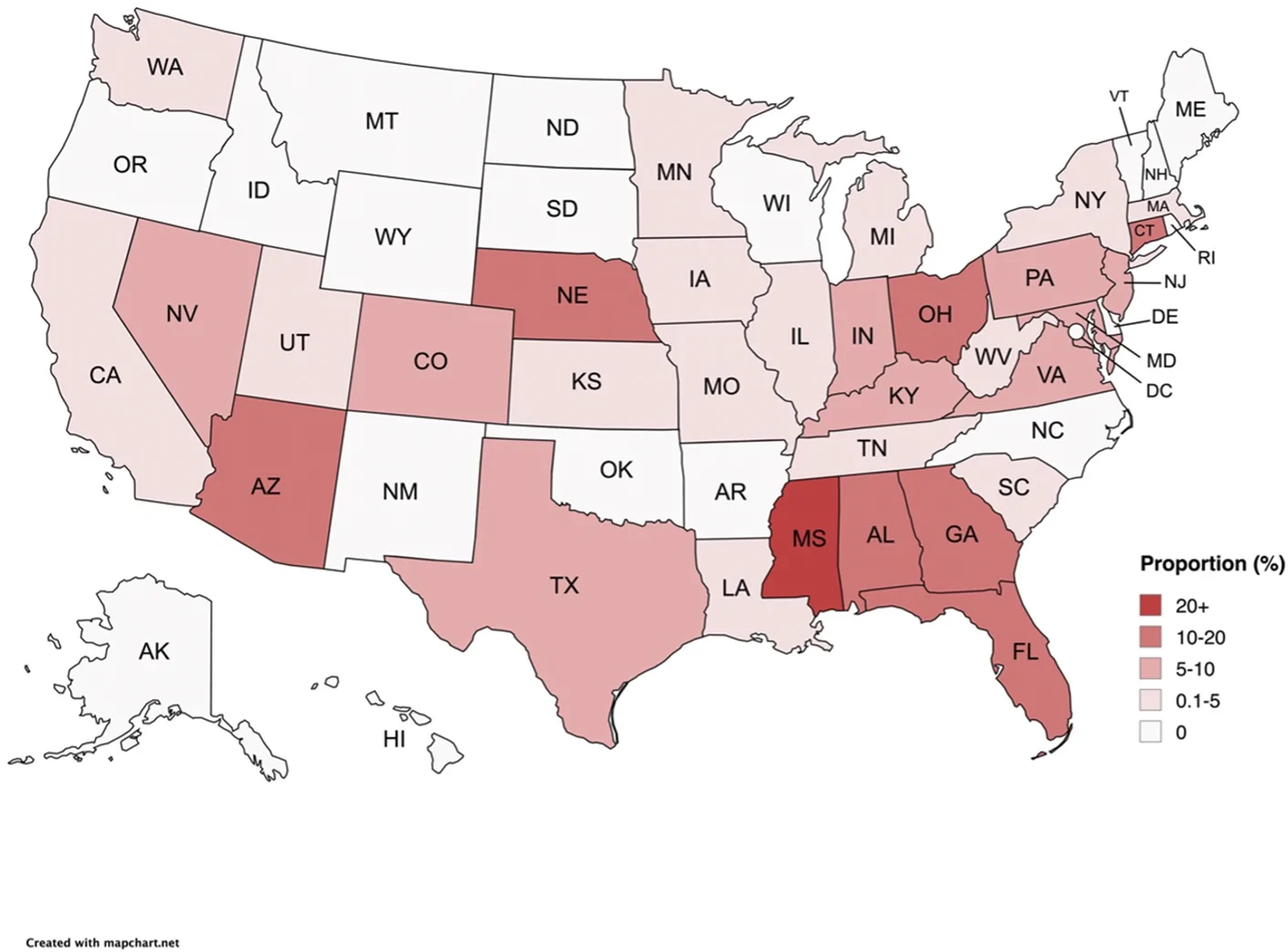
Proportion of orthopaedic surgeons with PE-affiliated practice by state in 2023.

**Table 2:** Top 8 PE-backed Orthopaedic Platforms

PE Firm	State	No. of Practices*	No. of Orthopaedic Surgeons	No. of Other Surgeons†	No. of Nonsurgeon Physicians	No. of APPs and Therapists	No. of Total Providers
OrthoAlliance	Indiana	3	37	10	14	70	131
	Ohio	9	72	21	20	154	267
	**Total**	12	109	31	34	224	398
United Musculoskeletal Partners‡	Colorado	1	32	3	11	269	315
	Georgia	2	89	12	14	86	201
	Texas	8	70	8	12	76	166
	**Total**	11	191	23	37	431	682
US Orthopaedic Partners	Alabama	4	53	6	20	78	157
	Louisiana	2	11	2	4	11	28
	Mississippi	4	58	1	4	60	123
	**Total**	10	122	9	28	149	308
HOPCo	Connecticut	1	21	7	4	18	50
	Florida	4	30	11	8	71	120
	Nevada	1	13	1	3	16	33
	Pennsylvania	2	51	22	32	99	204
	**Total**	8	115	41	47	204	407
Spire Orthopedic Partners	Connecticut	3	33	5	8	47	93
	Massachusetts	2	26	3	9	48	86
	New York	2	25	4	10	29	68
	**Total**	7	84	12	27	124	247
Orthopedic Care Partners	Arizona	1	18	7	10	60	95
	Colorado	1	15	0	5	31	51
	Florida	4	26	6	8	43	83
	Missouri	1	3	2	1	1	7
	**Total**	7	62	15	24	135	236
Aligned Orthopedic Partners	D.C.	1	11	1	0	19	31
	Maryland	3	33	7	3	43	86
	Virginia	1	8	3	5	23	39
	**Total**	5	52	11	8	85	156
American Orthopedic Partners	California	1	7	1	4	3	15
	Illinois	1	1	5	0	0	6
	New Jersey	2	13	0	7	18	38
	New York	1	3	2	0	4	9
	**Total**	5	24	8	11	25	68

APP = advanced practice providers, DC = District of Columbia, MD-PPAS = Medicare Data on Provider Practice and Specialty, PE = private equity, and TIN: Tax Identification Number.

Source: Authors’ calculation of data from PitchBook, MD-PPAS, and manual data collection.

* Practices were defined by individual TINs

† Surgeons classified as hand surgery, plastics and reconstructive surgery, neurosurgery, and podiatry in MD-PPAS data set

‡ United Musculoskeletal Partners purchased Novum Orthopedic Partners, and its 4 practices in Texas, in 2022

## Discussion

PE presence has expanded rapidly in the orthopaedic market. By 2023, nearly 1-in-20 orthopaedic surgeons were PE-affiliated across 32 states. This parallels the penetration rate seen at a larger scale in health care^1^. This is the first study to examine the characteristics of orthopaedic practices that have been acquired by PE entities. Several patterns were noted.

First, from 2020 to 2021, there was a 360% increase in PE acquisitions of orthopaedic practices. This immediately followed the COVID-19 pandemic. Several studies have demonstrated the financial ramifications of the pandemic on orthopaedists^26-28^. It is possible that because of financial losses from the decline in case volumes and elective procedures, orthopaedic groups were more willing to partner with a financial investor. In addition, interest rates were at record lows because of the pandemic, which made it easier for PE firms to acquire cheap debt to finance acquisitions^29^. Cardiology practices experienced similarly dramatic increases in PE investment following the pandemic^9,10^.

Several additional factors could have influenced the growth of PE in orthopaedics. For example, the period of rapid growth in investments overlaps with changes to the Medicare inpatient-only lists. Total knee replacements were removed from this list in 2018, and total hip replacements were removed in 2020^30,31^. With the option of performing these predictable, high-volume surgeries at ASCs, the opportunity for significant financial gains made orthopaedic practices even more attractive to PE groups^32,33^. Future research should examine whether PE-acquired practices increase the provision of these surgeries.

PE-acquired practices reflect a disproportionate share of certain subspecialties relative to the national landscape. Physicians in PE-acquired practices are more likely to practice sports medicine, arthroplasty, hand, spine, and foot and ankle compared with the relative national rates of these subspecialties^21^. Each of these subspecialties has procedures, or a rising proportion of procedures, that can be performed outpatient and in ASCs^34^. Furthermore, spine, arthroplasty, and sports medicine are the highest reimbursed subspecialties in the field^35,36^. Meanwhile, trauma, pediatrics, and tumor are underrepresented in PE-acquired groups. These specialties often manage larger proportions of publicly or underinsured patients while delivering care primarily at academic and tertiary care centers. It is likely that the breakdown of subspecialties seen in the PE-acquired groups also reflects what is seen in private practice orthopaedics overall.

One-in-10 providers in acquired practices were nonsurgical physicians, including PM&R, pain medicine, and anesthesiologists. This demonstrates the multidisciplinary character of many modern orthopaedic practices as they take on managing the full scope of musculoskeletal care. Meanwhile, more than half of providers in PE-acquired practices were ancillary providers, such as APPs and physical and occupational therapists. Ancillary services are well-established avenues of income for orthopaedists^37-39^. Elsewhere in health care, there have been demonstrated higher rates of ancillary providers following PE acquisition, as well^23^. Future research must examine whether PE acquisitions change the orthopaedic workforce composition, patient referrals, implications on resource utilization and healthcare spending, and the resulting effects on patient care, quality, and access.

Geographic analysis demonstrates predilection for certain regions and markets. Similar to the 2022 study of Boddapati et al., we found nearly half of all practice acquisitions occurred in the South^16^. At present, many states have a Corporate Practice of Medicine (CPOM) doctrine, that in theory, prevents nonlicensed individuals or entities from owning medical practices. However, based on prior research, there does not appear to be a connection between the presence of a CPOM doctrine and the growth of PE investments within a state^40^. This may be explained, in part, by reliance on MSOs to circumvent CPOM restrictions. For example, PE firms can hire a medical doctor to lead the clinical practice while using an MSO for all nonclinical endeavors^4,17^.

The present study has several limitations. First, while we used a combination of PitchBook and manual internet searches to compile our list of PE-affiliated orthopaedic practices, it is possible there was additional PE activity during the study period not captured in our data. We did not include any practices that were acquired by PE firms from 2024 to present. Furthermore, the definition of PE can vary, and there are no data sources that track deal structures with granularity, while many PE partnerships incorporate nondisclosure terms in the physician employment contracts. In our study, we defined it as leveraged buyouts of patient-facing clinics that are financially backed by a PE sponsor. A prior study used a broader definition of PE, which included real estate investment trusts, which could explain the variation in deal counts between our studies^16^. At this time, there is no systematic data source tracking deal structure, secondary transactions, bankruptcies, closures, or legal activity, so we are unable to comment on these potential occurrences following PE involvement in the orthopaedic setting. Another limitation comes from reliance on the MD-PPAS database. We rely on their reporting of TIN and NPI data while also using their PECOS subspecialty classifications. We did not have individual provider billing information, so when categorizing providers between surgeons and nonsurgeons, we relied on assumptions based on their given specialty classification. We did not have access to the 2023 MD-PPAS data set, so the 2022 version was used for 2023 acquisitions. It is also likely our national estimate of orthopaedic surgeons is undercounted because we did not include surgeons who had listed themselves as hand surgery in MD-PPAS, which includes orthopaedically trained hand surgeons. Future research must examine provider experiences, including physician turnover. Finally, we did not report demographic data, including age, gender, and race, of the providers in acquired practices, which could be informative in future work on this topic.

## Conclusion

PE investment in orthopaedics has expanded significantly in recent years with a decided increase from 2020 to 2021. By 2023, 4.8% of orthopaedic surgeons were in PE-backed practices, with substantial variation across states. This is the first study that has examined the characteristics of orthopaedic practices that have entered agreements with PE firms. In the practices that are targeted, there is overrepresentation of subspecialties with historically higher reimbursements (spine, arthroplasty, and sports medicine) and high outpatient surgical volume (sports medicine, arthroplasty, hand, and foot and ankle). Furthermore, there is great interest in practices that are multidisciplinary with heavy ancillary provider presence. Future work should examine the influence of PE affiliation on provider turnover, practice patterns, and clinical outcomes.

## Appendix

Supporting material provided by the authors is posted with the online version of this article as a data supplement at jbjs.org (https://links.lww.com/JBJSOA/B367). This content was not copyedited or verified by JBJS.
